# Children's strategies in drop-landing

**DOI:** 10.3389/fpsyg.2022.982467

**Published:** 2022-12-02

**Authors:** Rosa Angulo-Barroso, Blai Ferrer-Uris, Júlia Jubany, Albert Busquets

**Affiliations:** ^1^Institut Nacional d'Educació Física de Catalunya (INEFC), Universitat de Barcelona (UB), Barcelona, Spain; ^2^Department of Kinesiology, California State University, Northridge, CA, United States; ^3^Faculty of Health Sciences at Manresa, Universitat de Vic – Universitat Central de Catalunya (UVic-UCC), Manresa, Spain

**Keywords:** kinematics, kinetics, muscle activity, coordination, landing, land-run, young children

## Abstract

**Introduction:**

Landing is a critical motor skill included in many activities performed in the natural environment by young children. Yet, landing is critically relevance to ensure proper stability and reduce injury. Furthermore, landing is an integral part of many fundamental motor skills which have been linked to greater physical activity, sport participation, and perceived competence in children. Our aim was to examine the drop-landing strategies of young children focusing on the lower extremity with a multi-variant approach.

**Methods:**

Forty-four children divided into four age groups (G1:3–4.5 y, G2:4.5–6 y, G3:6–7.5 y, G4:7.5–9 y) performed 20 drop-land trials in four different conditions: predictable stationary landing, running to the left, to the right, and stay in place. Fifteen reflective markers, two force plates, and ten surface electromyography (sEMG) sensors were used to collect data. MANOVAs (Group x Condition) were conducted separately for the kinematic, kinetic, and sEMG variables.

**Results:**

Only significant group effects were found (kinematic MANOVA *p* = 0.039, kinetic MANOVA *p* = 0.007, and sEMG MANOVA *p* = 0.012), suggesting that younger groups (G1, G2) differed to the older groups (G3, G4). G1 showed less knee flexion and slower ankle dorsi-flexion during the braking phase compared to G3, while G2 presented smaller ankle dorsi-flexion at the braking phase and smaller ankle range of motion than G3. Overall kinetic variables analysis showed a group difference but no group differences for any single kinetic variable alone was found. Regarding sEMG, G1 during the flight phase exhibited longer tibialis anterior and hamstrings activity than G3 and G3 & G4, respectively; and an earlier start of the hamstrings' impact burst than G4. In addition, distal to proximal control was primarily used by all groups to coordinate muscle activity (in response to impact) and joint motion (after impact).

**Discussion:**

Perhaps a developmental critical point in landing performance exists at 4–5 years of age since G1 presented the largest differences among the groups. This suggests that to improve landing strategies could start around this age. Future studies should examine if playground environments that include equipment conducive to landing and practitioners in the kindergarten schools are adequate vehicles to empower this type of intervention.

## Introduction

The acquisition of proficiency in fundamental motor skills (FMS) such as running or throwing has been recognized as a building block for greater physical activity, play, sport participation, and better perceived competence (Okely et al., 2001; Stodden et al., 2008; Barnett et al., 2009). Most children continue developing these FMS until age twelve, then they go on to refining them as developmental changes progress into adulthood (Barber-Westin et al., 2006). FMS have been classified as locomotor and object manipulation (Magill and Anderson, 2014), with landing not being included in this traditional classification despite its common use (Seefeldt, 1986; Zhao et al., 2021). Some authors consider landing as a stabilizing motor skill (Mckinley and Pedotti, 1992) but not a FMS because landing typically occurs after a drop from a height or a jump; in other words, landing is considered a subsequent part of FMS such as running, jumping, and hopping. Regardless of its classification, it is clear that FMS and stabilizing motor skills (including landing) are part of the natural course of physical growth and development. Therefore, targeting these skills during practice would better equip children with proper technique and enhance their future physical activity potential since active children are more likely to be active adults (Okely et al., 2001; Barnett et al., 2009). Although landing is a critical motor skill included in many daily, recreational, and sport activities and its development seems to be interconnected to FMS, research focusing on the development of landing as a motor skill is scarce.

In addition to the relevant role that proper landing may play in the development and physical activity participation of a child, adequate technique in landing is important to prevent potential injuries. Most young children enjoy going to the playground, whether in school or at a park, and practice several motor skills including landing. Playground typical equipment includes swings, slides, elevated platforms, and monkey bars among others. Landing effectively from these devices is fundamental to safe participation in many play activities such as jumping from an elevated platform or dropping from a swing or monkey bar. Unfortunately, most injuries occur during these conditions. In fact, Loder (2008) studied children's injuries in the playground and found that injuries owning to the monkey bars remained the same from 1991 to 2005 while those owning to slides or swings decreased. The authors suggested that prevention strategies to reduce number of fractures should be directed at monkey bar equipment and landing surfaces. Besides these contextual characteristics affecting landing performance, children must adapt their motor skills to their constantly changing body and maturing nervous system. The greatest incidence of sprains and ACL injuries occurs during adolescence while landing (DiStefano et al., 2015) and it could be related to poor or incorrect landing acquisition during childhood. Taking together all these data may indicate the need for early interventions to ensure better landing skills and yet studies examining the development of landing motor strategies from younger ages (kindergarten and elementary school years) are very scarce.

Research focused on the development of landing mainly compared children around 9–10 years old and adults considering different types of landing. These types can be grouped on (1) drop-land tasks, which entails hanging from a bar or taking off from an elevated platform and landing (Hinrichs et al., 1985; Larkin and Parker, 1998; McMillan et al., 2010; Kim and Lim, 2014; Christoforidou et al., 2017; Estevan et al., 2020; Schroeder et al., 2021; Koo et al., 2022; Moir et al., 2022), and (2) jump-land tasks, which entails jumping horizontally or upwards (off a box or not) and landing (Hass et al., 2003; Russell et al., 2007; Xu et al., 2020). Despite of the possible differences related to the task specificity, previous literature presented evidence that pre-pubescent children between 7 and 12 years are less efficient diminishing the rate loading (higher peak of forces and shorter time to these peaks and also to the end of the braking phase) (Larkin and Parker, 1998; McKay et al., 2005; Lazaridis et al., 2010) because of: (1) their anticipatory strategies rely mostly on their muscle activation with higher time of activation before impact (Christoforidou et al., 2017); (2) during the impact they increase the muscle co-activity of the knee muscles but not the ones related to the ankle dorsiflexion (Croce et al., 2004; Russell et al., 2007; Wild et al., 2009), and (3) after impact, they tend to present more proximal-distal control with more muscle co-activity and limited range of motion of the lower limb joints in the sagittal plane (Hinrichs et al., 1985; Larkin and Parker, 1998; Hass et al., 2003; Croce et al., 2004; Kim and Lim, 2014; DiStefano et al., 2015; Raffalt et al., 2017; Niespodziński et al., 2021). To our knowledge, only Jensen et al. (1994) studied younger children (3–4 years old) in comparison with adults suggesting that children coordinate joint actions similarly to adults during landings after a vertical jump but exhibited a poor control of the muscle strength to perform adequate joint displacement and/or velocities.

When observing children in the playground, one realizes that children's landing actions are usually followed by another task (running, for example). Furthermore, these tasks are typically initiated in response to the changing context, which sometimes cannot be predicted because of the variable behavior of other children in the same space requiring a change of the initial direction to avoid bumping into each other. It is often mentioned that an unanticipated change of direction is a high risk movement associated with ACL injuries (Fuerst et al., 2017; Whyte et al., 2018; Weir et al., 2019) because the limited time to respond to the stimulus could lead to suboptimal decision making and errors in coordination that can promote injuries (Swanik et al., 2007; Yom et al., 2019). It is also plausible that individuals use alternative motor strategies to prepare landings with unanticipated response (Yom et al., 2019), injuries could appear when the alternative motor strategy is not good enough or is not properly used before starting movements following landing. The effects of an unanticipated change of direction movement to run from a double-leg landing are not well studied. Yom et al. (2019) reported no significant differences in vertical ground reaction forces and in hip, knee and ankle maximum flexion values when comparing adults performing anticipated or unanticipated change of directions to run after double-leg landings from a monkey bar. There is some research in children focusing on the combination of landing when it is followed by another predictable task (Croce et al., 2004; McKay et al., 2005; Swartz et al., 2005; Lazaridis et al., 2010; DiStefano et al., 2015; Niespodziński et al., 2021) but only, to our knowledge, Rosales et al. (2018) studied younger children (with typical development, TD, and with autism spectrum disorder, ASD) drop-landing followed by anticipated or unanticipated running conditions. The authors reported similar results in kinematics and muscle activity across conditions while more mature landing strategies were shown by TD with longer bursts of muscle activation during impact and shorter time to maximum knee and hip flexion during the braking phase.

Given the scarcity of evidence and the fact that effective motor skill performance like landing depends on previous early motor learning (Santello and Mcdonagh, 1998; Santello, 2005; Barber-Westin et al., 2006), it seemed reasonable to focus our research on the earlier years (3–9 years) which, at the same time, could provide new insights about the motor control development of landing. There are endless possibilities for the combinations of the aforementioned tasks. The self-initiate drop-land task seemed to be the simpler of all, and therefore most suitable to be studied in very young children. However, because landing rarely occurs in a vacuum, landing tasks that require to be followed by an unexpected or expected action are also relevant to understand overall landing strategies. The purpose of this study was to examine the drop-landing strategies of young children focusing on the lower extremity with a multi-variant approach (i.e., kinematic, kinetic, and muscle activity variables). We hypothesized that youngest children compared to older children will show a less efficient landing with (1) lower and shorter rate of loading due to impact; (2) less muscle specific pre-activation before impact but more co-contraction throughout the landing; (3) less flexion in the hips, knees, and ankles (dorsiflexion) during the landing; and (4) more use of proximal-distal sequences to coordinate motion and muscle activation.

## Methods

### Participants

Forty-four children (16 girls, 28 boys aged 3.1–8.9 y) participated in the study voluntarily (Table 1). The participants had no known history of lower extremity injuries. The participants were divided into four age groups: G1 (aged 3–4.5 y), G2 (aged 4.5–6 y), G3 (aged 6–7.5 y), and G4 (7.5–9 y). Subjects were not specialized in vertical jumps through training (e.g., volleyball, gymnastics) but they could participate in extracurricular activities. Children were recruited from schools in the San Fernando Valley and service learning programs at the university. Parents provided informed consent to participate in the study. The Institutional Review Board at California State University, Northridge, approved the study.

**Table 1:** General characteristics of the sample.

	**Group 1 (*****N** **=*** **11; 3** ♀**)**		**Group 2 (*****N** **=*** **10; 4** ♀**)**		**Group 3 (*****N** **=*** **10; 1** ♀**)**		**Group 4 (*****N** **=*** **13; 9** ♀**)**	
	**Mean**	**SD**	**Mean**	**SD**	**Mean**	**SD**	**Mean**	**SD**
Age (years)	3.95	0.49	5.31	0.33	6.70	0.33	8.42	0.32
Height (cm)	102.51	6.91	112.93	4.58	124.30	7.03	130.45	5.12
Weight (kg)	16.18	2.45	20.06	3.23	25.69	4.78	27.49	4.10
Leg lenght (cm)	42.44	4.07	50.26	2.63	55.95	5.36	60.07	3.05
Reach height (cm)	124.76	9.34	141.92	5.77	157.72	11.37	167.54	7.93
Maximum vertical jump (cm)	133.59	12.00	158.77	6.02	179.32	15.38	191.79	11.12
Bar height used (cm)	145.59	15.55	168.40	6.36	189.52	15.76	201.27	10.15

♀ = female birth sex.

### Procedure

**Figure 1:**
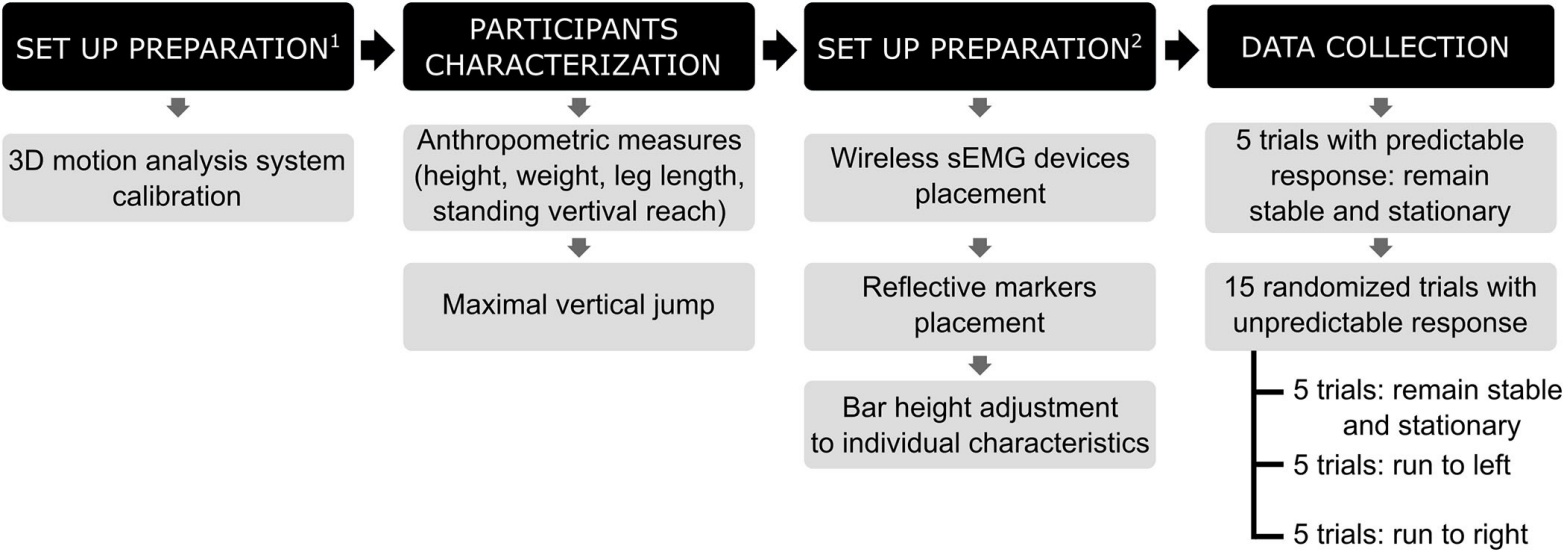
Flow chart of the experimental procedure.

**Figure 2:**
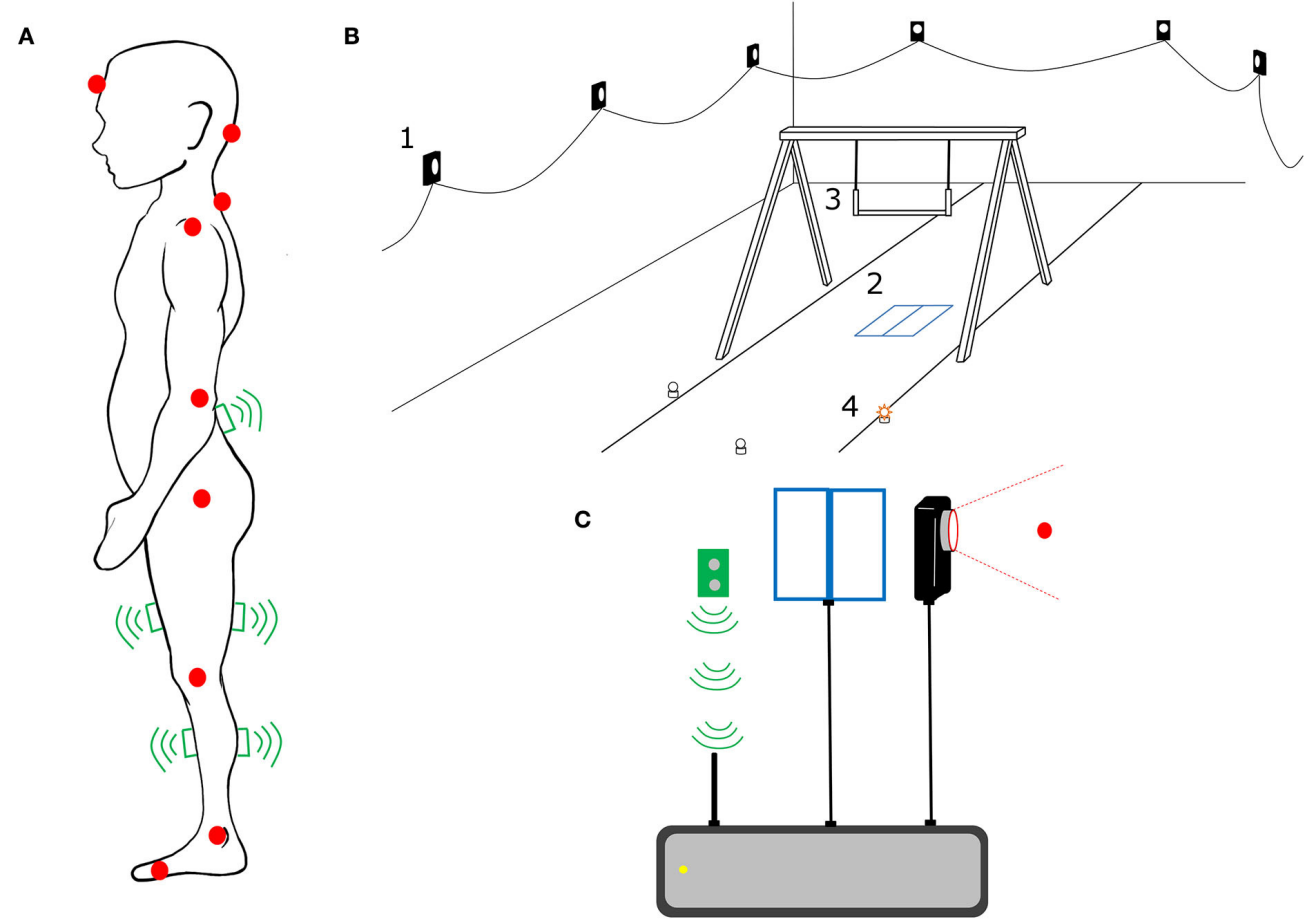
Schematic illustration of the experimental setup: **(A)** sketch of the reflective markers and the wireless surface electromyography (sEMG) devices disposition on participants; **(B)** infrared cameras to capture 3D motion data (2), two force plates (2), adjustable high bar (3), and light cues to respond during unpredictable response trials (4); and **(C)** flow chart of the 3D motion data from sEMG, force plate, and infrared cameras data synchronized through the specialized plug-ins and hardware of the Qualisys motion analysis system.

The participants and their caregivers came to the motor development laboratory once to carry out the whole experimental procedure (Figure 1). Participants were asked to change into compression shorts and a tank top and to remove their shoes. At the start of the session, anthropometric data were obtained, including: height, weight, leg length, and standing vertical reach. In addition, participant's maximal vertical jump was also measured. Next, a trained laboratory member placed 10 wireless surface electromyography (sEMG) devices (Delsys Incorporated, Natick, MA, USA) on participants after their skin was cleaned and abraded with an alcohol solution. sEMG was placed and recorded on both sides of the body for gastrocnemius (G), tibialis anterior (T), quadriceps (Q), hamstrings (H), and erector spinae (E), following the SENIAM guidelines (Hermens et al., 1999) (Figure 2A). In addition, 15 reflective markers were placed on the following anatomical landmarks: center of the forehead, base of the skull, cervical vertebrae 7 (C7), and on both sides for the acromion, lateral epicondyle of the humerus, greater trochanter, lateral side of the knee, lateral malleolus, and the fifth metatarsal (Figure 2A). Afterwards, participants performed the landing task consisting on landing from a monkey bar (Figure 2B). The individual bar height was determined following Rosales et al. (2018). For safety reasons, the individual bar height was tested for each child using few assisted drops before data collection. One of the researchers lifted the participants helping them to steadily hang on the bar at the start of each trial. Participants performed 20 trials of the landing task, where they were asked to land onto 2 force plates (Kistler, Winterthur, Switzerland) (Figure 2B); one foot on each force plate. Trials were executed in four different landing conditions. On one of the conditions, participants were totally aware of what they had to do upon landing (predictable response, P), while for the other three conditions participants had to respond to a light cue (stop light, light at their left, or light at their right side) lit by the force plates upon initial contact when landing (unpredictable response, U). Therefore, the four conditions were: a predictable response condition were participants had to land on their feet and remain stable and stationary (PS); an unpredictable response condition were participants had to land on their feet and remain stable and stationary (US); and two unpredictable response conditions were participants had to land and then run toward the light located to their left (UL) or right (UR) side. Each participant performed 5 trials of each condition, always starting with 5 non-randomized PS trials and, afterwards, performing the following 15 trials of the rest of the conditions presented in random order. For the PS and US trials, participants were instructed to “land and remain as still as possible for 5 s (if you see the stop light, for US)”, while for the UL and UR trials, participants were instructed to “land and run as fast as possible toward the lit light”. When a trial was considered not valid because participants dropped before the Go signal or because participants landed with both feet touching one force plate, an additional trial of that condition was performed. At least 2 min rests were provided every 5-trial set.

### Data collection

Kinematic data were captured at 100 Hz using a ten-camera Qualisys 3D motion analysis system (Qualysis AB, Göteborg, Sweden) (Figure 2B). Prior to data collection, the motion analysis system was calibrated according to the manufacturer's recommendations. The two force plates, embedded into the floor, were employed to capture ground reaction forces at a sampling frequency of 2,000 Hz. The muscle activity was recorded with 10 wireless Trigno Delsys sEMG sensors with the following characteristics: sampling frequency 1926 Hz, CMRR >110 dB at 50/60 Hz, gain: 1.000, bandwidth: 10–500 Hz, and bipolar surface Ag/AgCl disc electrodes (diameter: 0.8 cm, inter-electrode distance: 2 cm). Force plate and sEMG data were collected and synchronized along with the 3D motion analysis system through the specialized plug-ins and hardware of the Qualisys motion analysis system (Figure 2C).

### Data analysis

The objective of a successful land is to absorb the kinetic energy of the body, while refrain the lower extremity from collapsing under the force and to maintain balance and stability (Mckinley and Pedotti, 1992; Haywood and Getchell, 2014; Christoforidou et al., 2017). Most of the previous studies have divided landing in: (a) flight phase, which usually starts when the center of mass begin to descend and ends just prior to touch-down (also called impact); (b) pre-impact, a time window included in the flight phase typically defined from 100 ms prior to impact; (c) impact, as the instant of time of touch-down; (d) post-impact, a time window typically defined from impact to 100 ms later; and (e) braking phase, which starts the time from impact to maximum knee flexion and includes the post-impact window. Each of these divisions contributes differently to better understand changes of the motor strategies to a more mature landing execution. For example, the muscle activation before impact is critical to study anticipatory actions to face the impact, while the periods after impact allow us to focus on how muscle activation patterns and multi-joint movement coordination reduce the rates of loading until equilibrium is reached (Mckinley and Pedotti, 1992; Liebermann, 2008; McNitt-Gray, 2016).

Data analysis was performed with custom made Python scripts (Python Software Foundation) and in Labwindows CVI2010 (National Instruments, Austin, TX, USA) for the Kinematics, force plates, and sEMG data. A modified algorithm for detecting sEMG onset/offset following Jubany and Angulo-Barroso (2016) was also used for the sEMG data analysis.

**Figure 3:**
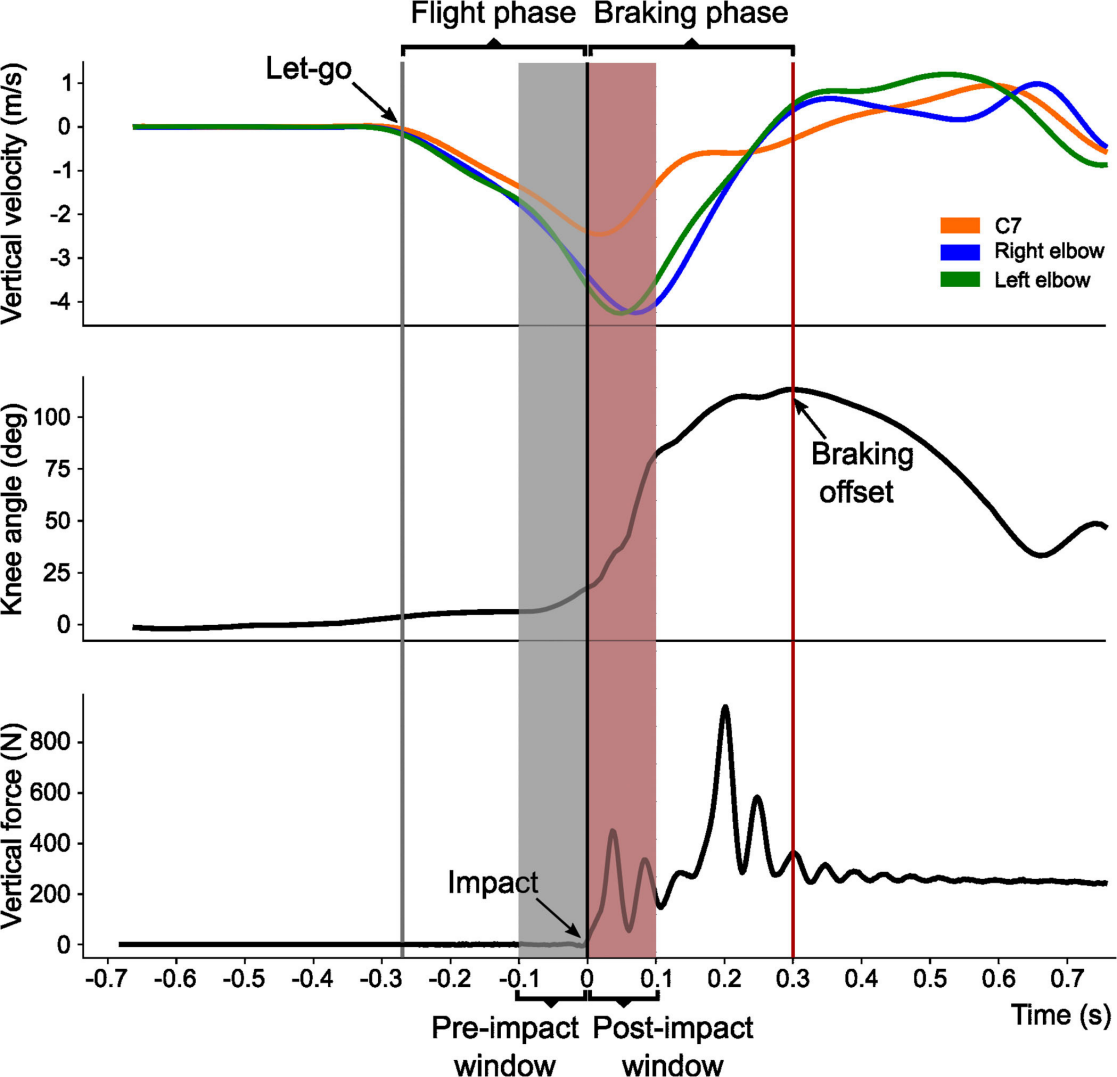
Example of kinematics and kinetics data with identification of the main events, phases, and windows utilized for the data analysis. Three events were characterized: (a) Let-go event, marking the instant of the bar release and defined from the vertical velocity of the C7, Right elbow, and Left elbow markers (gray vertical line); (b) Braking offset event, representing the end of the impact absorption and characterized through the knee angular position (red vertical line); and (c) Impact event, indicating the initial contact with the floor and identified using the vertical force data from the force plates (black vertical line). Two phases were defined from these events: the Flight phase, comprised between the Let-go and Impact events; and the Braking phase, comprised between the Impact and Braking offset events. In addition, a Pre-impact (gray shadowed area) and Post-impact (red shadowed area) 100 ms windows were defined around the Impact event.

Kinematics data were filtered with a recursive low-pass 4th-order Butterworth filter with a cut-off frequency of 6 Hz. From filtered kinematics data, vertical velocities of the C7, right elbow, and left elbow markers were computed. In addition, bilateral hip, knee, and ankle angular positions and angular velocities were also calculated. The impact event identified though the analysis of the force plates data (see impact event definition in the next paragraph) was set as zero on the timeline. Two additional events were identified though the kinematics data: the let-go event and the braking offset event (BO). Let-go was characterized as the latest time-point where a 2% of the maximum negative vertical velocity was obtained for the C7, right elbow, or left elbow markers at the start of their descending trajectory after the participants released the bar. The BO was defined as the latest time-point where the maximum right or left knee flexion (i.e., minimum knee angle) was obtained within a time window of a maximum of 350 ms after the impact event. These three events were used to define the flight phase (FP, from let-go event to impact event) and the braking phase (BP, from impact event to BO) which were used in the subsequent analyses. See Figure 3 for a graphical example where events and phases are identified.

Raw vertical force plate data were used to identify the following events. Impact (Imp) was defined as the earliest time-point were one of the two plates reached a vertical force of 10 N or greater. Afterwards, two peaks were identified in the vertical force registry of each plate: first peak after impact event (P1) and maximum peak force after the first peak (P2).

**Figure 4:**
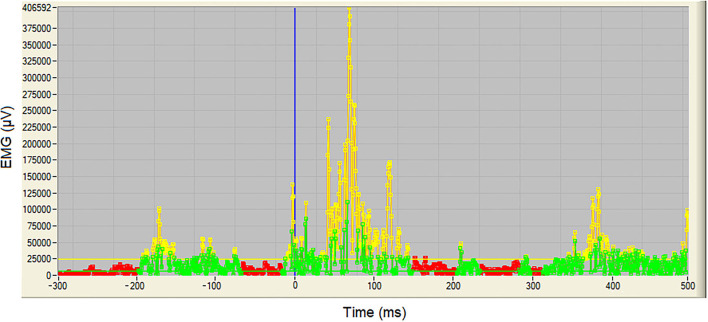
Example of a sEMG burst detection procedure with 300 ms prior to impact and 500 ms after impact. This detection involved two steps: (1) an initial detection identifying the continuous data that were higher than the mean plus 8 SD of the baseline values for at least 1.55 ms (3 data points) which yielded a temporary definition of the burst segment duration and its corresponding temporary onset and offset burst points (in yellow); and (2) a final onset and offset definition of the burst where burst onset and offset were redefined calculating the mean of a backwards or forward, respectively, running window (4.15 ms = 8 data points) around the temporary burst points defined in the first step and establishing the earliest or latest data point, respectively, at which the window mean was higher than the baseline mean plus 1 SD (in green). All data between the final burst onset and the final burst offset were considered as one sEMG burst. The rest of the data points were considered to be at baseline level (in red). The vertical blue line indicates the impact event (Imp).

Surface EMG was filtered using a recursive 4th-order band-pass Butterworth filter with 20–500 Hz cut-off frequencies. The impact event (Imp) defined through force plates data was used to label as time 0 and was transferred to all sEMG channels. The sEMG baseline signal for each muscle was taken as the lowest mean value (200 ms window) found across all valid trials for each participant when the participant was hanging from the bar (i.e., before the let-go event defined using kinematic data). Burst onsets and offsets were detected during the 300 ms prior and 500 ms after to impact event. This detection involved two steps: (1) an initial detection process identifying potential burst segments; and (2) a final onset and offset definition of the true bursts (see Figure 4 for more in depth explanation).

After individual muscle bursts were identified, two co-contraction burst pairs were defined; one between the tibialis anterior (T) and gastrocnemius (G) and the other between quadriceps (Q) and hamstrings (H). A co-contraction burst was identified when simultaneous activity bursts were previously identified for both muscles. For sEMG variables only, and in addition to the already defined flight and braking phases, two 100 ms time-windows were defined around impact: pre-impact (−100 to 0 ms) and post-impact (0 to +100 ms).

### Variables

#### Kinematic variables and flexion timing pattern

Kinematic variables were computed during the BP for each valid trial. They were calculated as the mean of the values extracted from the right and left side joint angle (full extension 0°) and angular velocities data: maximum hip and knee flexion and ankle dorsi-flexion angle defined as the maximum angle (θ_Hip_max_, θ_Kne_max_, and θ_Ank_max_, respectively); range of motion defined as the difference between the maximum and minimum joint angles (θ_Hip_max−min_, θ_Kne_max − min_, and θ_Ank_max−min_); time of maximum flexion angle for the hip (*t*θ_Hip_max_), knee (*t*θ_Kne_max_) and ankle (*t*θ_Ank_max_); maximum flexion velocity for the hip (ω_Hip_max_), knee (ω_Kne_max_), and ankle (ω_Ank_max_). Additionally, the time of the maximum flexion angles (*t*θ_Hip_max_, *t*θ_Kne_max_, and *t*θ_Ank_max_) were utilized to characterize the joint flexion timing pattern of the lower limb. Patterns where typified as: Distal (Dis_kinPattern) when the ankle dorsiflexion preceded the knee and hip maximum flexions (distal to proximal flexion), Proximal (Pro_kinPattern) when the hip maximum flexion preceded the knee and ankle maximum flexion (proximal to distal flexion), Mixed (Mix_kinPattern) when in the sequence the maximum knee flexion is the last to occur (e.g., ankle < hip < knee), and Other (Oth_kinPattern) for any other possible combinations (e.g., ankle = Knee < hip).

#### Kinetic variables

Kinetic variables were computed using vertical force data and after defining Imp, P1, P2, and BO (the latter defined through the kinematics data) on each side (right and left). All variables extracted from the force data registry characterized the BP of each valid trial and they were calculated as the mean of both sides and body weight was used to normalize force values and calculations from them. The following dependent variables were obtained: force value at peak one (*F*_P1_), force value at peak two (*F*_P2_), time of peak two (*tF*_P2_), impulse from impact to peak two (*I*_Imp−P2_), and impulse from impact to braking offset (*I*_Imp−BO_).

#### Muscle activity variables and muscle patterns response to the impact

Regarding the sEMG variables, percentage of active time (muscle % activity = time of burst activity within phase or window/total phase duration ^*^ 100) of each muscle (E, G, H, Q, and T) was computed for the FP and the pre-impact window (Pre). In addition, % activity of co-contracting muscles pairs (T-G and Q-H) was computed for the post-impact window (Pos) and the BP (pair % co-contracting activity = time of co-contraction activity within phase or window/total phase duration ^*^ 100). Means of both sides (right and left) for each valid trial were calculated to define the muscle activity (E_%_FP_, E_%_Pre_, G_%_FP_, G_%_Pre_, H_%_FP_, H_%_Pre_, Q_%_FP_, Q_%_Pre_, T_%_FP_, T_%_Pre_,) and the co-contraction activity variables (Q-H_%_BP_, Q-H_%_Pos_, T-G_%_BP_, T-G_%_Pos_). On the other hand, anticipatory or reactive muscle activity to impact was characterized identifying the occurrence of impact and post-impact bursts. Impact bursts were defined as those muscle bursts that started at least 40 ms before impact and lasted, at least, 40 ms after impact. Post-impact bursts were defined as the first activity burst that started 40 ms after the impact or later and that lasted 80 ms or more. Impact bursts were used to compute the percentage of occurrence of an impact activity burst (%B_Imp_) along the five trials of each condition (E_%B_Imp_, G_%B_Imp_, H_%B_Imp_, Q_%B_Imp_, and T_%B_Imp_). The start-time of those impact bursts (tB_Imp_) was also obtained for each trial and muscle. The mean between the tB_Imp_ of both sides (right and left) in each trial were used to calculate E_tB_Imp_, G_tB_Imp_, H_tB_Imp_, Q_tB_Imp_, and T_tB_Imp_. Finally, the start-times of the impact and post impact bursts were used to examine which first two muscles initiated their activation in response to the impact. If an impact burst was not found for a muscle, the post-impact burst was used instead. Based on the first two activated muscles, six muscle activation onset patterns where typified: distal (Dis_EMGPattern) when G and T were the two first muscles activated; proximal (Pro_EMGPattern) when H and Q were the two first muscles activated; mixed anterior (Mix_Ante__EMGPattern) when Q and T were the two first muscles activated; mixed posterior (Mix_Post__EMGPattern) when G and H were the two first muscles activated; and mixed crossed (Mix_Cros__EMGPattern) when H and T or G and Q are the two first muscles activated, and other (Oth_EMGPattern) any other possible combinations when E was activated.

### Statistical analysis

General lineal mixed model with repeated measures in each variable were conducted to assess the trial effect, except for the percentage of impact activity burst occurrence variables (E_%B_Imp_, G_%B_Imp_, H_%B_Imp_, Q_%B_Imp_, and T_%B_Imp_) which were calculated using the five trials of each condition. General lineal mixed model were applied because non-normal distribution of data was detected with Shapiro–Wilks tests and missing measurements appeared in some participants' trials (Bolker et al., 2009; Harrison et al., 2018). Two models were calculated, the first where children were included as a random factor while trial, condition, and age group were introduced as fixed factors. In the second model, no random factors were included. The model without random factors demonstrated better fit after checking the Bayesian Information Criteria. This model showed no significant trial effect in 59 out of 66 variables. Given that mostly no significance trial effects were found, individual means for each variable were calculated across the five trials for each condition (P, US, UL, UR).

Five MANOVAs with repeated measures (4 Age groups x 4 Conditions) were used to evaluate age and condition effects and interactions on kinematics, kinetics, flight phase sEMG, braking phase sEMG, and burst occurrence variables. Due to different sample size of the impact bursts start-time of each muscle, age-group and condition effects were assessed using ANOVAs with repeated measures (4 Age groups x 4 Conditions) for each variable (E_tB_Imp_, G_tB_Imp_, H_tB_Imp_, Q_tB_Imp_, and T_tB_Imp_). Potential Type I error was controlled adjusting p values from univariate ANOVAs conducting Bonferroni's correction. Also, sphericity-corrected values by Greenhouse-Geisser were obtained when appropriate. Finally, pairwise comparisons with Bonferroni correction were used to establish differences between age-groups and conditions. The effect size was measured by partial eta squared (small effect size: η^2^*p* ≤ 0.010; medium effect size: η^2^*p* ≤ 0.059; large effect size: η^2^*p* ≤ 0.138).

Chi-square contingency tables were computed to evaluate the effect of the age and the conditions in the joint flexion timing pattern and muscle activation onset pattern responses to the impact. Bonferroni correction was applied to adjust z test calculation for each row.

Statistical significance of all tests was set at the *p* < 0.05 level and only statistically significant results were reported. All statistical tests were performed with SPSS PASW Statistics 21 software (SPSS, Inc., Chicago, IL, USA).

## Results

Kinematics, kinetics, flight phase sEMG, braking phase sEMG, and burst occurrence variables were compared by MANOVAs with age-group as a between factor and the four task conditions as within factor. Results from these MANOVAs showed significant Group main effects on the kinematics, the kinetics, and the flight phase sEMG variables (Table 2). No significant Condition main effects or Group x Condition interactions were found. Subsequent one-way ANOVAs from MANOVAs that presented Group main effects revealed significant differences in four kinematic variables (θ_Ank_max_, θ_Kne_max−min_, θ_Ank_max−min_, and ω_Ank_max_) and three flight phase sEMG variables (G_%_FP_, H_%_FP_, and T_%_FP_) (Table 2). On the other hand, ANOVAs conducted on muscle impact bursts start-time showed significant Group and Condition main effects on G_tB_Imp_ and H_tB_Imp_ but a significant interaction only was found in H_tB_Imp_ (Table 3). Finally, joint flexion timing pattern and muscle activation onset pattern responses to the impact yielded statistically significant differences by group when chi-square contingency tables were computed (Tables 4, 5).

**Table 2:** Significant main effects and Group x Condition interactions: MANOVAs in **bold**-*italicized* and subsequent ANOVAs significant results.

**Variable group**	**Variable name**	**Main effect or interaction**	***F***	***df***	***p***	***η^2^*p**	**Power**	**Pairwise comparisons**	**Homogeneous subsets**	
									**1**	**2**
***Kinematics***		**Group**	***1.598***	***3,39***	**0.039**	***0.39***	***0.981***			
	θ_Ank_max_	Group	3.280	3,39		0.201 0.031	0.706	G2 > G3	G1, G2, G4	G1,G3, G4
	θ_Kne_max−min_	Group	3.339	3,39	0.029	0.204	0.715	G1 < G3	G1, G2, G4	G1,G3, G4
	θ_Ank_max−min_	Group	3.986	3,39	0.014	0.235	0.796	G2 < G3	G1, G2, G4	G3, G4
	ω_Ank_max_	Group	3.38	3,39	0.028	0.206	0.721	G1 > G3	G1, G2, G4	G2, G3, G4
***Kinetics***		**Group**	***2.306***	***3,39***	**0.007**	***0.253***	***0.97***			
***sEMG: % activation***		**Group**	***1.873***	***3,39***	**0.012**	***0.369***	***0.990***			
	G_%_FP_	Group	3.173	3,39	0.035	0.196	0.690	-	G1, G2, G3, G4
	H_%_FP_	Group	4.973	3,39	0.005	0.277	0.884	G1 > G3, G4	G1, G2	G2, G3, G4
	T_%_FP_	Group	4.017	3,39	0.014	0.236	0.800	G1 > G4	G1, G2, G3	G2, G3, G4

θ_Ank_max_, ankle maximum angle (i.e., maximum flexion); θ_Kne_max−min_, knee flexion range of motion; θ_Ank_max−min_, ankle dorsi-flexion range of motion; θ_Ank_min_, ankle maximum angular velocity (i.e., maximum flexion velocity); G_%FP, Gastrocnemius percentage of activation during the flight phase; H_%FP, Hamstrings percentage of activation during the flight phase; T_%FP, Tibialis anterior percentage of activation during the flight phase; G1, group 1 (aged 3-4.5 years); G2, group 2 (aged 4.5-6 years); G3, group 3 (aged 6-7.5 years); G4, group 4 (aged 7.5-9 years).

**Table 3:** Significant main effects and Group x Condition interactions ANOVAs for sEMG burst variables.

**Variable**	**Main effect or interaction**	***F***	***df***	***p***	***η^2^*p**	**Power**	**Pairwise comparisons**
G_tB_Imp_	Group	4.516	3,39	0.046	0.184	0.651	-
	Condition	2.926	3,39	0.005	0.104	0.874	PS < UR
H_tB_Imp_	Group	3.157	3,39	0.035	0.195	0.688	G1 < G4
	Condition	5.582	3,39	0.003	0.125	0.898	PS < UL
	Group x Condition	2.569	3,39	0.016	0.165	0.881	US: G1 < G3, G4
							G3: PS < UL, UR, US

G_tB_Imp_, start-time of the gastrocnemius impact burst; H_tB_Imp_, start-time of the hamstrings impact burst; G1, group 1(aged 3–4.5 years); G2, group 2 (aged 4.5–6 years); G3, group 3 (aged 6–7.5 years); G4, group 4 (aged 7.5–9 years); PS, predictable landing response condition were participants had to land on their feet and remain stable and stationary; UL, unpredictable landing response condition were participants had to land on their feet and then run toward the light located to their left; UR, unpredictable landing response condition were participants had to land on their feet and then run toward the light located to their right; US, unpredictable landing response condition were participants had to land on their feet and remain stable and stationary.

**Table 4:** Chi square contingency table for joint flexion timing pattern variables.

**Variable**	**Group**	**Dis_kinPattern**	**Pro_kinPattern**	**Mix_kinPattern**	**Oth_kinPattern**	**χ^2^**	***p***
		***n (%)***	***n (%)***	***n (%)***	***n (%)***		
Flexion timing patterns						47.24	< 0.001
	G1	167 (75.2)	3 (1.4)	16 (7.2)	36 (16.2)		
	G2	157 (78.5)	2 (1.0)	25 (12.5)	16 (8.0)		
	G3	131 (65.8)^*^	3 (1.5)	42 (21.1)^*^	23 (11.6)		
	G4	224 (85.5)^*^	1 (0.4)	12 (4.6)^*^	25 (9.5)		

Dis_kinPattern, the ankle dorsiflexion preceded the knee and hip maximum flexions (distal to proximal flexion); Pro_kinPattern, the hip maximum flexion preceded the knee and ankle maximum flexion (proximal to distal flexion); Mix_kinPattern, when in the sequence the maximum knee flexion is the last to occur; Oth_kinPattern, other possible combinations; G1, group 1 (aged 3-4.5 years); G2, group 2 (aged 4.5-6 years); G3, group 3 (aged 6-7.5 years); G4, group 4 (aged 7.5-9 years).

^*^Indicates significant *post-hoc* result after Bonferroni correction.

**Table 5:** Chi square contingency table for muscle patterns in response to the impact event.

**Variable**	**Group**	**Dis_EMG** **Pattern**	**Pro_EMG Pattern**	**Mix_Ante__EMG** **Pattern**	**Mix_Post__EMG Pattern**	**Mix_Cros__EMG** **Pattern**	**Oth_EMG Pattern**	**χ^2^**	***p***
		***n (%)***	***n (%)***	***n (%)***	***n (%)***	***n (%)***	***n (%)***		
Muscle patterns response to impact								119.02	< 0.001
	G1	96 (43.2)	0 (0.0)	8 (3.6)	53 (23.9)^*^	51 (23.0)^*^	14 (6.3)		
	G2	59 (29.5)^*^	3 (1.5)	34 (17.0)^*^	12 (6.0)^*^	69 (34.5)	23 (11.5)		
	G3	93 (46.7)	1 (0.5)	10 (5.0)	17 (8.5)	70 (35.2)	8 (4.0)		
	G4	103 (39.3)	2 (0.8)	11 (4.2)	41 (15.6)	88 (33.6)	17 (6.5)		

Dis_EMGPattern, gastrocnemius and tibialis anterior were the two first muscles activated; Pro_kinPattern, hamstrings and quadriceps were the two first muscles activated; Mix_Ante__EMGPattern, quadriceps and tibialis anterior were the two first muscles activated; Mix_Post__EMGPattern, gastrocnemius and hamstrings were the two first muscles activated; Mix_Cros__EMGPattern, hamstrings and tibialis anterior or gastrocnemius and quadriceps are the two first muscles activated; Oth_EMGPattern, other possible combinations when erector spinae was activated; G1, group 1 (aged 3-4.5 years); G2, group 2 (aged 4.5-6 years); G3, group 3 (aged 6-7.5 years); G4, group 4 (aged 7.5-9 years).

^*^Indicates significant post-hoc result after Bonferroni correction.

### Kinematic variables and flexion timing pattern

**Figure 5:**
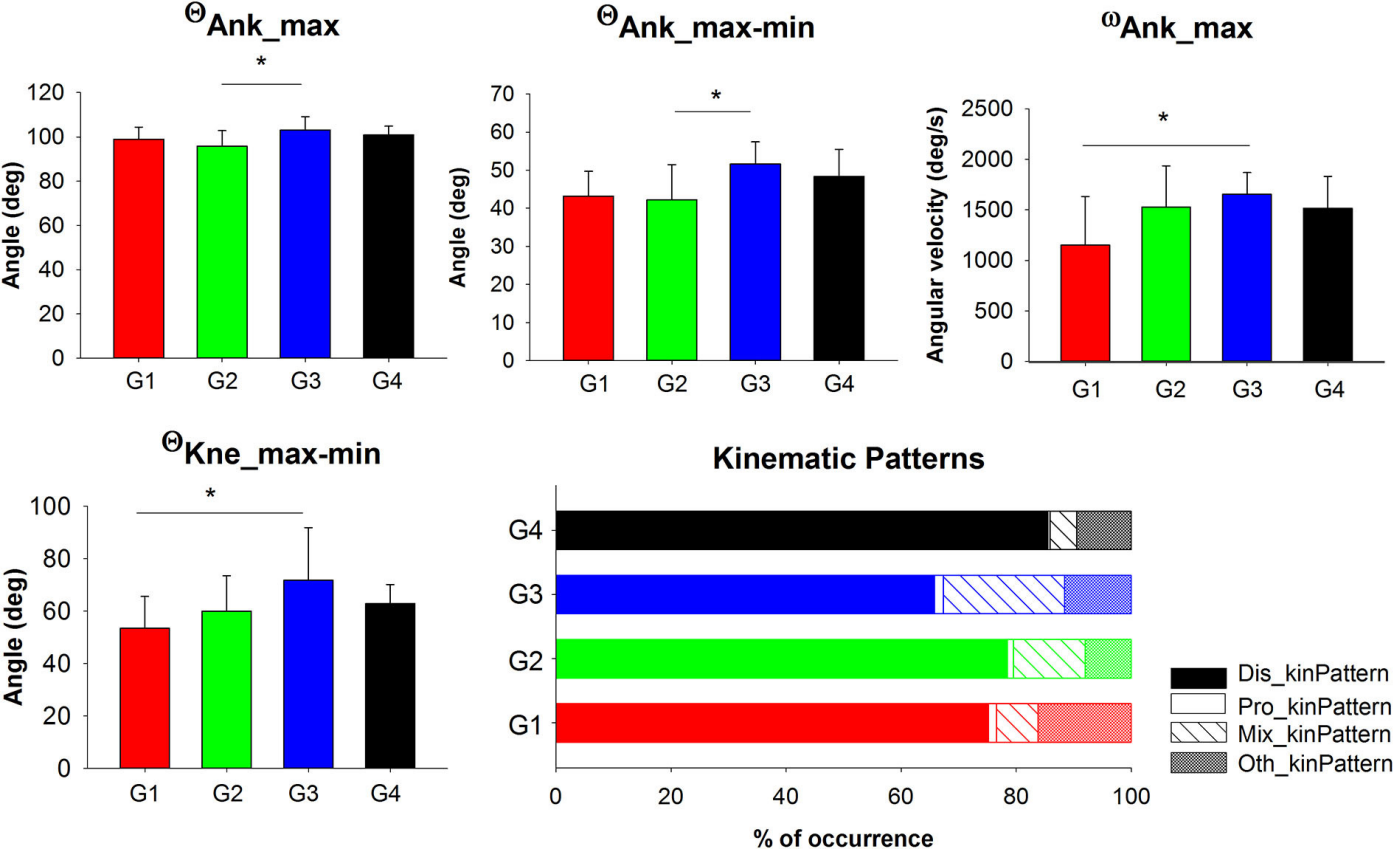
Significant Group main effects of kinematic variables and occurrence of the kinematic patterns. Mean and standard deviations of the kinematic variables and percentage of occurrences of the kinematic patterns were plotted by group. G1, group 1 (aged 3–4.5 years); G2, group 2 (aged 4.5–6 years); G3, group 3 (aged 6–7.5 years); G4, group 4 (aged 7.5–9 years); θ_Ank_max_, ankle maximum angle (i.e., maximum flexion); θ_Kne_max−min_, knee flexion range of motion; θ_Ank_max−min_, ankle dorsi-flexion range of motion; ω_Ank_max_, ankle maximum angular velocity (i.e., maximum flexion velocity); Dis_kinPattern, the ankle dorsiflexion preceded the knee and hip maximum flexions (distal to proximal flexion); Pro_kinPattern, the hip maximum flexion preceded the knee and ankle maximum flexion (proximal to distal flexion); Mix_kinPattern, when in the sequence the maximum knee flexion is the last to occur; Oth_kinPattern, other possible combinations. Asterisks (*) represent significant differences in the *post-hoc* analyses.

ANOVAs and their *post-hoc* showed differences between the third age group (G3) and the younger groups (G1 and G2), especially in the ankle motion variables (Table 2 and Figure 5). Concretely, the G3 performed larger ankle dorsi-flexion range of motion (θ_Ank_max−min_) compared to G2 by achieving lower values of maximum ankle dorsi-flexion (θ_Ank_max_). On the other hand, the youngest group (G1) performed the ankle dorsi-flexion range slower and flexed less the knee than G3. The differences presented and the homogeneous subsets created statistically by the MANOVA (Table 2) indicated that younger groups (G1, G2) performed drop-landings similarly to each other, but differently to the older groups (G3 and G4) which shared similarities but to a lesser extent.

Regarding the joint flexion timing pattern, all groups performed the drop-landings mainly with a distal sequence (Dis_kinPattern), specially the oldest group (G4) who showed the highest frequency (Table 4). Interestingly, G3 showed the lowest frequency using the distal sequence (Dis_kinPattern) while they executed the mixed pattern (Mix_kinPattern) more often (Figure 5).

### Kinetic variables

**Figure 6:**
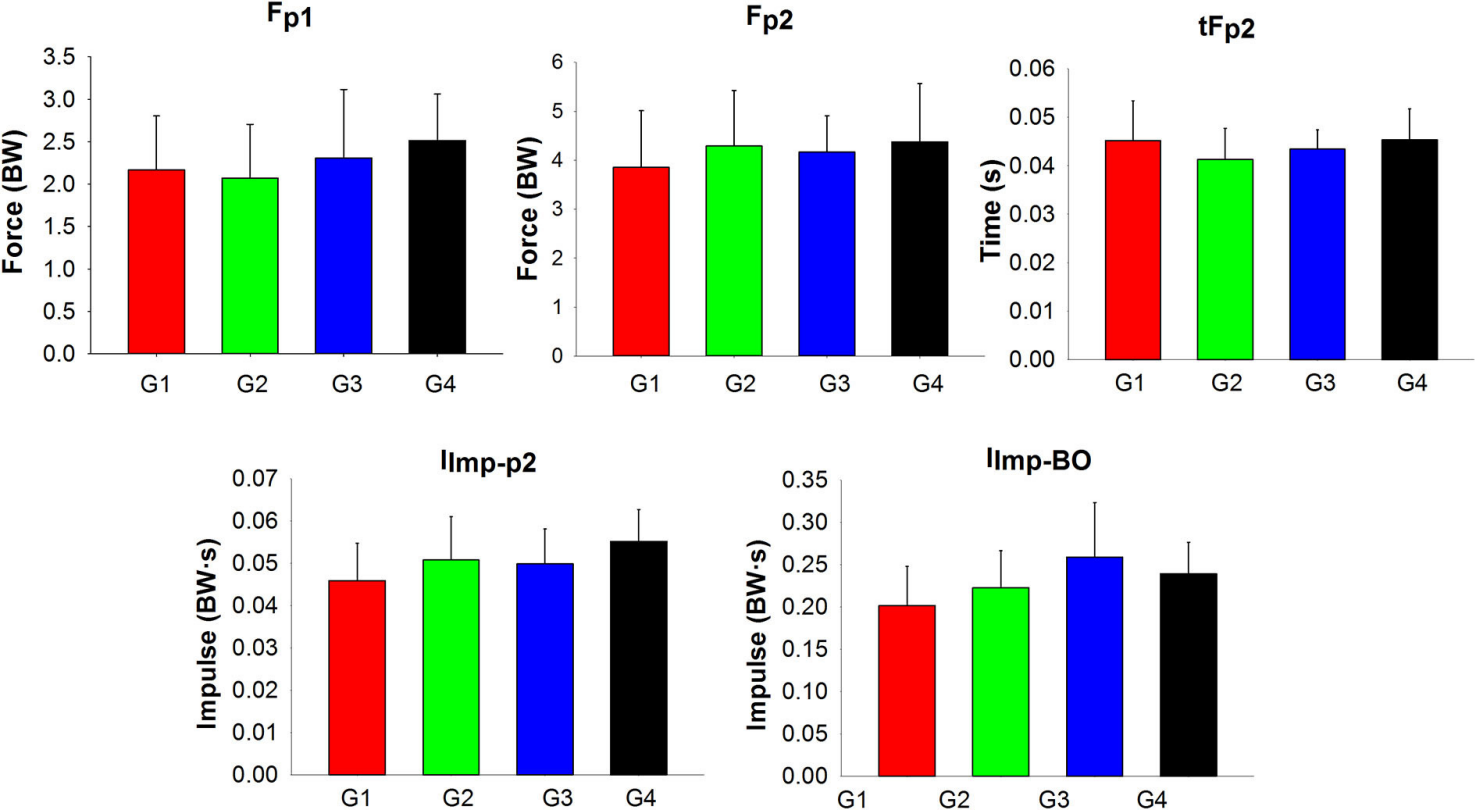
Group means and standard deviations of the kinetic variables. Despite a significant MANOVA Group effect for the kinetic parameters, no significant main group effects were found in the subsequent ANOVAs. G1, group 1 (aged 3–4.5 years); G2, group 2 (aged 4.5–6 years); G3, group 3 (aged 6–7.5 years); G4, group 4 (aged 7.5–9 years); F_P1_, force value at peak one; F_P2_, force value at peak two; tF_P2_, time of peak two; I_Imp−P2_, impulse from impact to peak two; and I_Imp−BO_, impulse from impact to braking offset.

Despite MANOVA results showed significant group main effect in the kinetic variables, no significant differences were found between groups when subsequent ANOVAs were conducted (Figure 6).

### Muscle activity variables and muscle patterns response to the impact

**Figure 7:**
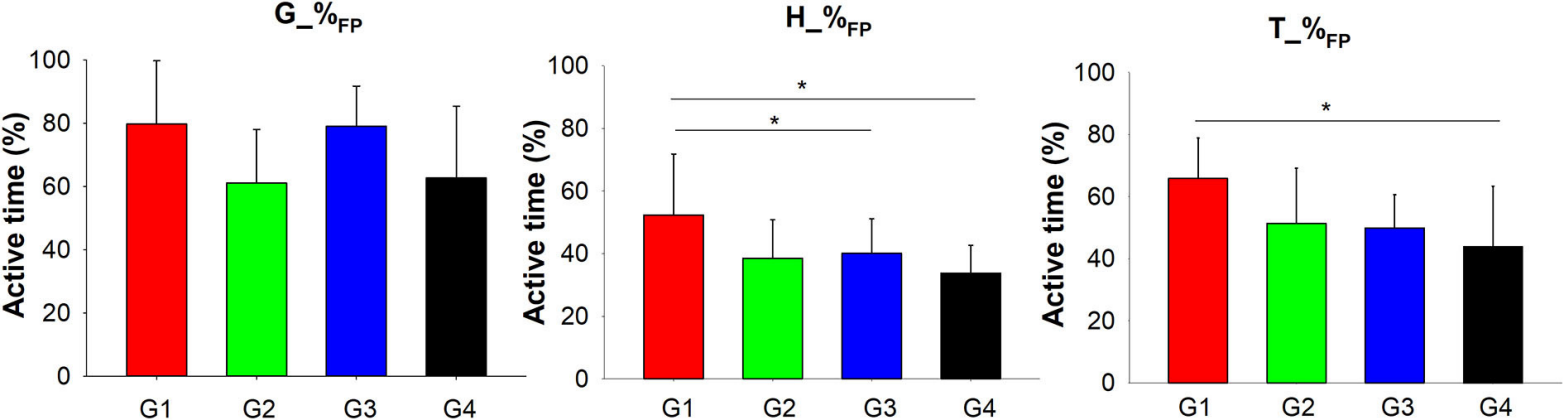
Significant Group main effects of sEMG variables. Mean and standard deviations were plotted by group. G1, group 1 (aged 3–4.5 years); G2, group 2 (aged 4.5–6 years); G3, group 3 (aged 6–7.5 years); G4, group 4 (aged 7.5–9 years); G_%_FP_, Gastrocnemius percentage of activation during the flight phase; H_%_FP_, Hamstrings percentage of activation during the flight phase; T_%_FP_, Tibialis anterior percentage of activation during the flight phase. Asterisks (*) represent significant differences in the *post-hoc* analyses.

ANOVAs and the *post-hoc* conducted on flight phase sEMG variables showed that the youngest group (G1) activated for more of the FP their hamstrings (H_%_FP_) than the older groups (G3 and G4) and their tiabialis anterior (T_%_FP_) than G4 (Table 2 and Figure 7). No pairwise differences were shown by the *post-hoc* analyses for the gastrocnemius percentage of activation during flight (G_%_FP_) although a group main effect was found by the ANOVA (Table 2 and Figure 7).

**Figure 8:**
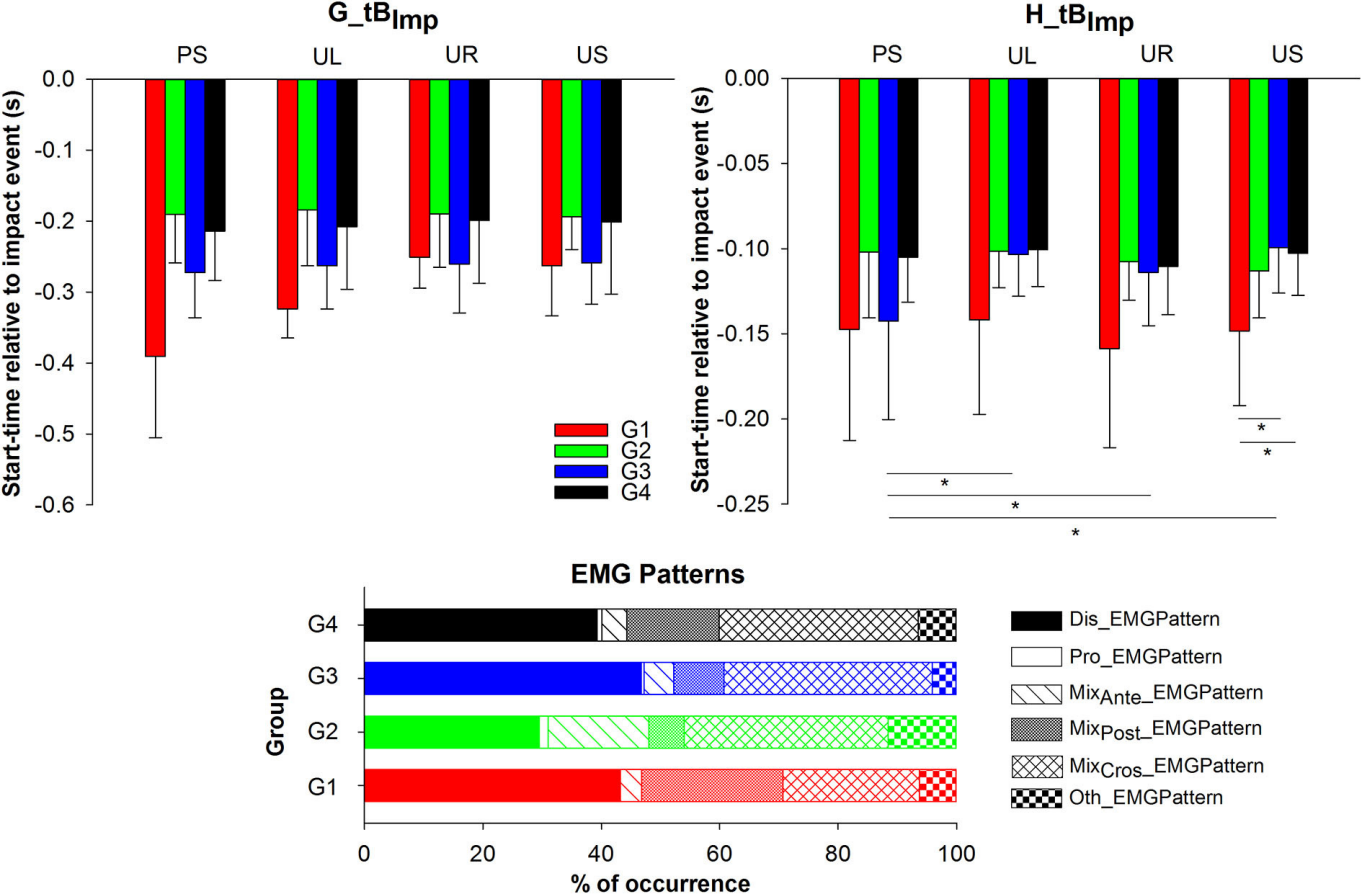
Significant Group x Condition interaction effects of the sEMG variables and occurrence of the muscle patterns in response to the impact event. Mean and standard deviations were plotted by condition and group and percentage of occurrences of the muscle patterns were plotted by group. G1, group 1 (aged 3–4.5 years); G2, group 2 (aged 4.5–6 years); G3, group 3 (aged 6–7.5 years); G4, group 4 (aged 7.5–9 years); G_tB_Imp_, start-time of the gastrocnemius impact burst; H_tB_Imp_, start-time of the hamstrings impact burst; PS, predictable stay condition; UL, unpredictable left condition; UR, unpredictable right condition; US, unpredictable stay condition; Dis_EMGPattern, gastrocnemius and tibialis anterior were the two first muscles activated; Pro_kinPattern, hamstrings and quadriceps were the two first muscles activated; Mix_Ante__EMGPattern, quadriceps and tibialis anterior were the two first muscles activated; Mix_Post__EMGPattern, gastrocnemius and hamstrings were the two first muscles activated; Mix_Cros__EMGPattern, hamstrings and tibialis anterior or gastrocnemius and quadriceps are the two first muscles activated; Oth_EMGPattern, other possible combinations when erector spinae was activated. Asterisks (*) represent significant differences in the *post-hoc* analyses.

Regarding the muscle impact bursts start-time, ANOVAs yielded significant differences between groups for the gastrocnemius and hamstrings but *post-hoc* comparisons only showed an earlier hamstring burst performance by younger group (G1) in contrast to the oldest (G4) (Table 3 and Figure 8). In addition, gastrocnemius and hamstrings impact burst start-times during drop-landings with predictable response (PS) were significantly earlier than unpredictable responses (UR and UL, respectively) (Table 3 and Figure 8). A Group and Condition interaction was also found for H_tB_Imp_ (Table 3) showing that for the US drop-landings the youngest group (G1) initiate the hamstring burst earlier than older groups (G3 and G4) (Table 3 and Figure 8) and that G3 activated earlier the hamstrings during the impact of the predictable drop-landings (PS) than the non-predictable trials (UL, UR, and US) (Table 3 and Figure 8).

Regarding the muscle patterns response to the impact, the distal sequence (Dis_EMGPattern) was mostly used by all groups but the second most frequent sequence used differed across groups (Table 5). Thereby, older groups (G3 and G4) showed higher frequencies performing mixed crossed sequences (Mix_Cro__EMGPattern) while G1 and G2 presented higher frequency of the mixed anterior sequence (Mix_Ant__EMGPattern) and the mixed posterior sequence (Mix_Pos__EMGPattern), respectively.

## Discussion

This multi-variant study explored how young children modified their motor strategies to drop and land across age (from 3 to 9 years) and the possible effect of landing followed by an unexpected or an expected action. Our results indicated that younger children (G1: 3–4.5 and G2: 4.5–6 years) seemed to modulate differently their drop-landing strategies but with similar effectiveness than the older groups (G3: 6–7.5 and G4: 7.5–9 years). In addition, children did not generally modify their drop-landing strategies to adapt them to unexpected or expected follow-up actions. These results only partially supported our hypothesis that less flexion would have been performed by the youngest children during the landing. Nonetheless, our study provided new evidence to the scarce literature on how landing develops during kindergarten and primary school ages using an ecological task design.

During the FP and preparing for the impact, the youngest children (G1) showed longer muscle activity of the tibialis anterior (ankle dorsi-flexor) and hamstrings (knee flexor, hip extensor) than older children (both muscles for G3, only hamstrings for G4) and earlier start of the hamstrings' impact burst than the oldest group (G4). It is proposed that muscle activation before touchdown plays a major role on the individual landing strategy. An anticipatory strategy usually presented by less experienced performers is to increase joint stiffness by increasing the antagonist muscle activity before impact (Liebermann, 2008; Wild et al., 2009; Christoforidou et al., 2017). Previous studies showed hamstrings activity in pre-pubescents [8–10 years in Russell et al. (2007); 7–8 years in Wild et al. (2009)] and an increase of the tibialis anterior activity in untrained girls (9–12 years) (Christoforidou et al., 2017) to prepare the landing. Our results seemed to indicate that the youngest children pre-activated the antagonist muscles for a longer time likely to increase joint stiffness, especially the knee, and increase passive absorption of kinetic energy.

In addition, these longer and earlier antagonist muscle activities previous to impact observed in the youngest children could also indicate differences in the learning of the control mode of the landing task. Upon landing, the onset and duration of the voluntary muscle activity seems to be mainly planned using a feedforward mode of control, which estimates the instant of impact based on the height of fall and/or the time of flight (Santello, 2005; Liebermann, 2008). Learning of this feedforward mode of control should be influenced by age and experience (Schmitz et al., 2002; Croce et al., 2004; Quatman et al., 2006; Lazaridis et al., 2010; Christoforidou et al., 2017). In fact, untrained pre-pubescent girls (9-12 years) showed shorter muscle pre-activation compared to gymnasts when exposed to increasing drop heights, which was considered as indicative of a worst estimation of the instant of impact and its consequences. Also, Rosales et al. (2018) reported that typically developing children between 3 and 4.5 years presented longer muscle bursts in comparison to children with autism spectrum disorder of the same age indicating a poor feedforward control of this last group. In our study, the height of the monkey bar was established for each child based on individual's anthropometrics and vertical jump ability (Rosales et al., 2018) and was constant across trials. Given that the height of fall was related to the height of jumps capability, it is possible that children from 4.5 years had a similar refinement of the feedforward control mode but not the youngest children (3.5–4.5 years) who had an underlearned control mode, characterized by earlier and longer antagonistic activation of hamstrings in comparison to the older groups. However, to properly study the consequences of the time to impact estimation and the feedforward control learning across ages, future studies changing the height of the bar relative to the anthropometrics characteristics and the vertical jump ability of the child are needed.

Regarding muscle coordination, time patterns of the muscle activation analyzed in this study yielded that the distal sequence was the most used for all age groups, while the proximal sequence did almost not occur. Interestingly, age-groups differed in their second most frequent sequences pointing out that children across age did not always activate muscles in the same sequence to prepare the landing. Concretely, children between 3–4.5 years relied more on performing sequences where anterior muscles of the leg (tibialis anterior and quadriceps) were the first muscles activated, while posterior muscles (gastrocnemius and hamstrings) were the two first muscles activated by children between 4.5–6 years. On the other hand, older groups (children >6 years) showed sequences where anterior and posterior muscle activations were mixed as their second most frequent muscle activity sequence. These results seemed to be partially in agreement with Jensen et al. (1994) hypothesis that timing relationship is more related to task demands (i.e., jump and land in their article) than performer skills obtained by age and/or experience. However, using a sequence where anterior or posterior muscles are the first muscles activated could be related to the estimated postural position at impact. This seemed to be clearer for the youngest children group who appeared to estimate impact with a posteriorized posture and thus they activated first the anterior leg muscles, or the second younger group, whom estimated an anteriorized posture and activated posterior leg muscles. Therefore, timing of the muscle activation would be related to task demands but also to estimation of the impact posture.

In general, no differences were found in the FP as a consequence of the knowledge or not of the task to do after landing, except for children between 6 and 7.5 years that showed earlier activation of the hamstring for the predictable response after landing trials in comparison to the non-predictable response trials. We did not expect differences between conditions when preparing the landing because cues to respond were activated after touching the force plates. In addition, previous studies in children (Rosales et al., 2018) and adults (Yom et al., 2019) using similar experimental conditions indicated that participants did not modify their flight phase regardless of the task to perform after landing. Surprisingly, G3 pre-activated earlier the hamstrings when they were aware that they had to remain stable and stationary after landing. It could be that to ensure the achievement of the task they further anticipated the time onset of the agonistic muscle activity and in consequence the stiffness of the knee.

After the impact, no differences in the muscle co-activity were observed across age groups but results seemed to indicate that younger children moved differently. The younger groups performed the absorption of the impact with less angular displacement and slower angular velocities in comparison to G3. In concrete, children between 3 and 4.5 years flexed less the knees and exhibited slower ankle dorsi-flexion during the braking phase, while smaller ankle dorsi-flexion and ankle range of motion was observed for the children between 4.5 and 6 years. It is suggested that a more efficient and mature landing performer will use multi-joint flexion of the leg and trunk to increase the duration of the post-landing period (corresponding to our braking phase) and then actively dissipate kinetic energy within a longer time period (McKay et al., 2005; Liebermann, 2008; Estevan et al., 2020). Our results are consistent with studies that proposed that less skilled children landing presented poor control of the ankle dorsiflexion (Hinrichs et al., 1985; Mckinley and Pedotti, 1992). In addition, results of our study appeared to indicate that children from 4.5 years of age already used a knee angular motion to modulate the impact effects as oldest children did. Given that all children groups presented similar co-contraction activity durations, it could be suggested that the observed differences in the knee and ankle joint motion were not so related to the control of the muscle activation but more related to the capacity to generate and control eccentric and explosive forces required to brake the landing (Jensen et al., 1994; Hass et al., 2003; Waugh et al., 2013). On the other hand, it is fair to notice that younger groups presented differences with the children of 6–7.5 years but not the oldest ones (7.5–9 years). When examining the group characteristics, the oldest group did not present the same linear change in the maximum vertical jump skill than the observed in the other age groups, while they maintained a similar linear growth as the rest of the groups. This disparity between the physical and the motor skill development could indicate that participants included in the oldest group were not as motor advanced as it would be for their age.

Furthermore, the analyzed time patterns of the joint motion after landing showed that children coordinated movements mostly using a distal sequence, while a proximal sequence was almost not used by children. Children between 6 and 7.5 years presented the lowest frequency using the distal sequence and highest use of timing patterns where the maximum knee flexion was the last to occur. Also, the older group used more frequently the distal sequence and used less the mix sequence. Regardless of these differences, our results agreed with Jensen et al. (1994) that mainly observed similar timing relationship of joint motion across ages. As the authors proposed previously it is plausible that these time patterns were related to task demands (i.e., jump and land in their article) but not necessarily to age and/or experience.

Despite the differences found during the flight and braking phases, similar kinetic values were obtained by all the age-groups demonstrating equivalent efficiency absorbing the impact. It is recommended that when the objective is to better understand developmental differences in landing strategies, it is critical to consider the relative height from which participants are required to land (task demands) (Weinhandl et al., 2015; Rosales et al., 2018). Therefore, the design of this study took extra care to control for the level of task demand and established the height to fall according to the anthropometric characteristics and the jumping ability of the child. In addition, all kinetic variables were normalized by body weight. Considering both factors (level of task demand adjustment and normalized values) could be the reason why our results did not show the age differences presented by other studies (Liebermann, 2008; Lazaridis et al., 2010; Iida et al., 2012). We recommend to compare studies using absolute different falling heights with caution and to design studies with the level of the task demand adjusted by anthropometrics and jumping ability whenever possible.

Predictable and unpredictable response after landing conditions did not affect the motor strategies performed by children during the BP. Similar results were reported for children (3–4.5 years) (Rosales et al., 2018) and adults (Yom et al., 2019) performing the same task. Our data could indicate that children between 3 and 9 years of age are not able to integrate landing and a subsequent task since we found no significant condition effects for any age group. However, taking into consideration that adults also presented the same behavioral results and that it was impossible to anticipate the response to the cue (it appeared when one foot touched the force plate), it seems reasonable to think that children did not respond to the unanticipated cues with a different or modified motor strategy.

Taking all together, evidences of this study could establish the bases from which to design physical activity session to enhance landing acquisition during childhood. We would suggest to primarily focus on intervention exercises that help to learn the feedforward control mode necessary to adequately estimate the instant of impact and, also, to improve the capacity of ankle, knee, and hips muscles to control and generate eccentric and explosive force to actively dissipate the kinematic energy instead of using muscle co-activity to adopt a stick strategy. In addition, description of the motor strategies could assist professionals to identify motor patterns of landing in non-typical developed children populations and lead possible interventions.

Regarding the contributions of this study to understand how drop-landing strategies developed during childhood and the use of relatively large sample, it has to be recognized that results are based on a cross sectional design and, then, no true developmental trajectories can be stablished. We did our best creating a set up for drop-landing similar to the one in the playgrounds; we assumed limited ecological validity data because they were collected in an experimental laboratory in favor of ensuring quality data. We only evaluated the sEMG on/off patterns and times of muscle activity to minimize the differential effects of electrode placement, movement artifact, and normalization technique. However, the proper use of the sEMG magnitude could have added information about the muscle activity level. Coordination in this study was assess using discrete outcome variables; a more accurate approach to analyze how children coordinate drop-landing would take in consideration all kinematic or sEMG measurements trajectories as a function of time. Since no differences in the motor strategies were found between unpredictable and predictable conditions in drop-landing tasks in children, it could be possible that differences appear in the motor response to the cue with longer time response. Studies to analyze motor response to unpredictable cues are needed. Researchers could design them with longer times that ensure capturing the initiation of the response and providing new insight in whether and how children modulate drop-landing strategies, both before and after initial contact with the floor. Finally, we assumed symmetry between the two legs and no sex-based differences and, maybe, some developmental achievements to perform landing are related to individual laterality or sex. Further researches are needed to cover all the above limitations but also comparisons of participants across lifespan are necessary to support our results, to assess targeted interventions or new playgrounds and kindergarten designs adapted to the youngest children characteristics.

## Conclusions

In summary, our results in young children (3–9 years) suggest that drop-landing strategies were related to age or task demands but not to the predictability of the task following the land. During the FP, the youngest children (3–4.5 years) showed longer antagonist leg muscle activity likely to increase the joint stiffness or maybe because they had an underlearned feedforward control mode to estimate the instant of impact. After the impact, children between 3 and 6 years showed a poor ankle dorsiflexion while knee flexion values were smaller only for the youngest children. These results together with the lack of muscle co-activity differences across age groups could indicate a reduced capacity to control and generate the adequate amount of eccentric and explosive force to actively dissipate the kinematic energy. In addition, all children showed a preference to use a distal sequence coordinating muscle activation to prepare the impact and coordinating joint motion after the impact, while a proximal sequence was rarely used. These timing relationship results suggested that coordination could be related to task demands but not to age and/or experience. On the other hand, the differences in the second most frequent muscle activation used during the FP could be an indication that impact posture estimation could modulate the pre-impact muscle coordination. Taken all results together, children between 3 and 6 years used different drop-landing strategies than older children (6–9 years) but with similar effectiveness. The largest differences presented by the youngest group could indicate that a developmental critical point in landing performance exists at 4–5 years of age. We would suggest to start targeted practice and interventions around this age together with studies examining the feasibility to conduct them in playgrounds and kindergarten environments.

## Data availability statement

The raw data supporting the conclusions of this article will be made available by the authors, without undue reservation.

## Ethics statement

The studies involving human participants were reviewed and approved by Institutional Review Board at California State University, Northridge (CA, USA). Written informed consent to participate in this study was provided by the participants' legal guardian/next of kin.

## Author contributions

RA-B contributed with the conception of the idea, design of the study, and the implementation of the research. JJ and RA-B contributed by providing sEMG burst analysis methodology and the custom made software to implement it. AB and BF-U contributed with the data treatment, variables calculation, and statistical analyses. All authors contributed to data analysis and writing of the manuscript.

## Funding

This study was supported by a small grant from the California State University, Northridge (CSUN), the Institut Nacional d'Educació Física de Catalunya (INEFC), Universitat de Barcelona (UB), Faculty of Health Sciences of Manresa, Universitat de Vic – Universitat Central de Catalunya (UVic-UCC), Manresa, Spain, and the Grup de Recerca en Activitat Física i Salut (GRAFiS, Generalitat de Catalunya 2017SGR/741).

## Conflict of interest

The authors declare that the research was conducted in the absence of any commercial or financial relationships that could be construed as a potential conflict of interest.

## Publisher's note

All claims expressed in this article are solely those of the authors and do not necessarily represent those of their affiliated organizations, or those of the publisher, the editors and the reviewers. Any product that may be evaluated in this article, or claim that may be made by its manufacturer, is not guaranteed or endorsed by the publisher.
